# Environmental Enteric Dysfunction Includes a Broad Spectrum of Inflammatory Responses and Epithelial Repair Processes

**DOI:** 10.1016/j.jcmgh.2015.12.002

**Published:** 2015-12-11

**Authors:** Jinsheng Yu, M. Isabel Ordiz, Jennifer Stauber, Nurmohammad Shaikh, Indi Trehan, Erica Barnell, Richard D. Head, Ken Maleta, Phillip I. Tarr, Mark J. Manary

**Affiliations:** 1 Genome Technology Access Center, Department of Genetics, Washington University School of Medicine, St. Louis, Missouri; 2 Department of Pediatrics, Washington University School of Medicine, St. Louis, Missouri; 3 Department of Community Health, College of Medicine, Blantyre, Malawi; 4 Children’s Nutrition Research Center, Baylor College of Medicine, Houston, Texas

**Keywords:** Environmental Enteropathy, Fecal Transcriptome, Stunting, Intestinal Inflammation, dHAZ, change in height-for-age z score, EED, environmental enteric dysfunction, FARMS, factor analyses for robust microarray summarization, G-CSF, granulocyte colony–stimulating factor, HAZ, height-for-age z score, IRON, iterative rank order normalization, KEGG, Kyoto Encyclopedia of Genes and Genomes, mRNA, messenger RNA, %L, lactulose permeability, qPCR, quantitative polymerase chain reaction, RMA, robust multi-array average

## Abstract

**Background & Aims:**

Environmental enteric dysfunction (EED), a chronic diffuse inflammation of the small intestine, is associated with stunting in children in the developing world. The pathobiology of EED is poorly understood because of the lack of a method to elucidate the host response. This study tested a novel microarray method to overcome limitation of RNA sequencing to interrogate the host transcriptome in feces in Malawian children with EED.

**Methods:**

In 259 children, EED was measured by lactulose permeability (%L). After isolating low copy numbers of host messenger RNA, the transcriptome was reliably and reproducibly profiled, validated by polymerase chain reaction. Messenger RNA copy number then was correlated with %L and differential expression in EED. The transcripts identified were mapped to biological pathways and processes. The children studied had a range of %L values, consistent with a spectrum of EED from none to severe.

**Results:**

We identified 12 transcripts associated with the severity of EED, including chemokines that stimulate T-cell proliferation, Fc fragments of multiple immunoglobulin families, interferon-induced proteins, activators of neutrophils and B cells, and mediators that dampen cellular responses to hormones. EED-associated transcripts mapped to pathways related to cell adhesion, and responses to a broad spectrum of viral, bacterial, and parasitic microbes. Several mucins, regulatory factors, and protein kinases associated with the maintenance of the mucous layer were expressed less in children with EED than in normal children.

**Conclusions:**

EED represents the activation of diverse elements of the immune system and is associated with widespread intestinal barrier disruption. Differentially expressed transcripts, appropriately enumerated, should be explored as potential biomarkers.

**Summary**

The host transcriptome in feces was characterized in 259 rural Malawian children at risk for environmental enteric dysfunction. A broad range of immune activation and defects in cell adhesion were found, coupled with decreased mucin expression, elucidating the pathobiology of this condition.

Stunting, defined as a height-for-age z score (HAZ) of less than -2, affects 26% of all children younger than the age of 5 years worldwide.^1^, ^2^ Stunting is associated with reduced neurocognitive capability, diminished immunocompetence, 20% of disability-adjusted life years lost in this age group, and more than 2.1 million deaths annually.^2^

Optimal gut health encompasses effective dietary nutrient absorption and a mucosal immune response that confines microbes to the lumen without inducing chronic tissue inflammation. Environmental enteric dysfunction (EED) is an asymptomatic, diffuse villous atrophy of the small bowel associated with chronic mucosal T-cell infiltration and reduced paracellular integrity.^3^ EED is highly prevalent, often without gastrointestinal symptoms, in poor children in the developing world.^4^, ^5^

EED typically is assessed with a dual sugar permeability test, whereby mannitol (molecular weight, 182 daltons) and lactulose (molecular weight, 342 daltons) are ingested under controlled conditions and quantified in the urine. Both sugars are neither degraded in the upper gastrointestinal tract nor systemically metabolized after absorption, and are excreted rapidly in the urine.^6^ Lactulose is a disaccharide, which can be absorbed only by passively crossing disrupted cell junctions, and thus the amount of this sugar in the urine reflects small-bowel permeability.^6^, ^7^ Mannitol, a monosaccharide, is absorbed across cell membranes and between cell junctions and is included to normalize lactulose uptake and excretion to the mucosal surface area, and to control for variations in gastric emptying time. Both the ratio of urinary lactulose to mannitol and the fraction of lactulose that is excreted in the urine (lactulose permeability [%L]) have been used to assess gut health. The dual sugar assay, although imperfect, is a theoretically sound measurement test of gut health.^7^

Much has been learned about health and disease in the past decade by using agnostic surveys of the human transcriptome,^8^ including, in recent years, reliance on RNA deep sequencing to profile transcriptional response to injury. Unfortunately, these methods have required RNA samples larger than 1 μg that have been processed to remove inhibitors of nucleic acid hybridization and nonhuman RNA. This requirement has limited our understanding of the host transcriptome analyses of feces from individuals.

This report details the development and application to a human cohort of a novel RNA selective isolation procedure from human feces, coupled with high-density, whole human transcriptome microarray technology to interrogate samples from 259 rural Malawian children with varying states of EED.

## Methods

### Study Design

This was a prospective cohort observational study of rural African children at high risk for EED. The primary outcomes were the correlation between %L and expression levels of protein coding genes, based on data that %L correlates with linear growth in this population.^9^, ^10^ Secondary outcomes were associations with Kyoto Encyclopedia of Genes and Genomes (KEGG) and canonical pathways in EED.

### Eligible Subjects

The study was conducted in rural Malawi, where populations practice subsistence farming (corn and beans), and reside in mud and thatch homes. Water is collected from boreholes and wells; electricity is unavailable. Inclusion criteria consisted of subjects between 12 and 61 months of age who reside in 1 of 6 rural communities under research surveillance, and included 810 children in total.^11^, ^12^, ^13^ This included a spectrum of children with EED, from no EED to severe EED. Children were excluded if they had a chronic disability or disease, severe acute malnutrition, or were receiving therapy for tuberculosis. All subjects were interviewed and examined by a physician and found to be free of pathologic conditions. Weight, length, and mid-upper-arm circumferences were measured by trained and monitored staff to determine nutritional status.

### Dual Sugar Absorption Testing

Dual sugar permeability testing was conducted in a supervised setting, and complete consumption of the sugars and collection of all urine during the subsequent 6 hours was verified.^12^ Children consumed no food or drink for 8 hours before drinking 20 mL of water into which 1 g of mannitol and 5 g of lactulose were dissolved. This was administered immediately after children voided. A dual sugar permeability test was considered successfully completed when all urine was collected for at least 4 hours after ingestion of the sugars, without spillage of dosing sugars or urine. Urine volumes were measured using a graduated cylinder, and a 2-mL aliquot was flash-frozen and shipped to the Baylor College of Medicine (Houston, TX) where urinary lactulose was measured using high-pressure liquid chromatography.^14^, ^15^ EED severity was assigned using population data from a larger clinical study such that the children with %L less than 0.2 were designated as not having EED, and those with %L greater than 0.2 and less than 0.7 were designated as having intermediate EED, and those with %L greater than 0.7 were designated as having severe EED.^9^ The transformation log_2_ (%L*100) was used to determine linear correlations between %L and microarray data.

### Stool Collection

**Figure 1:**
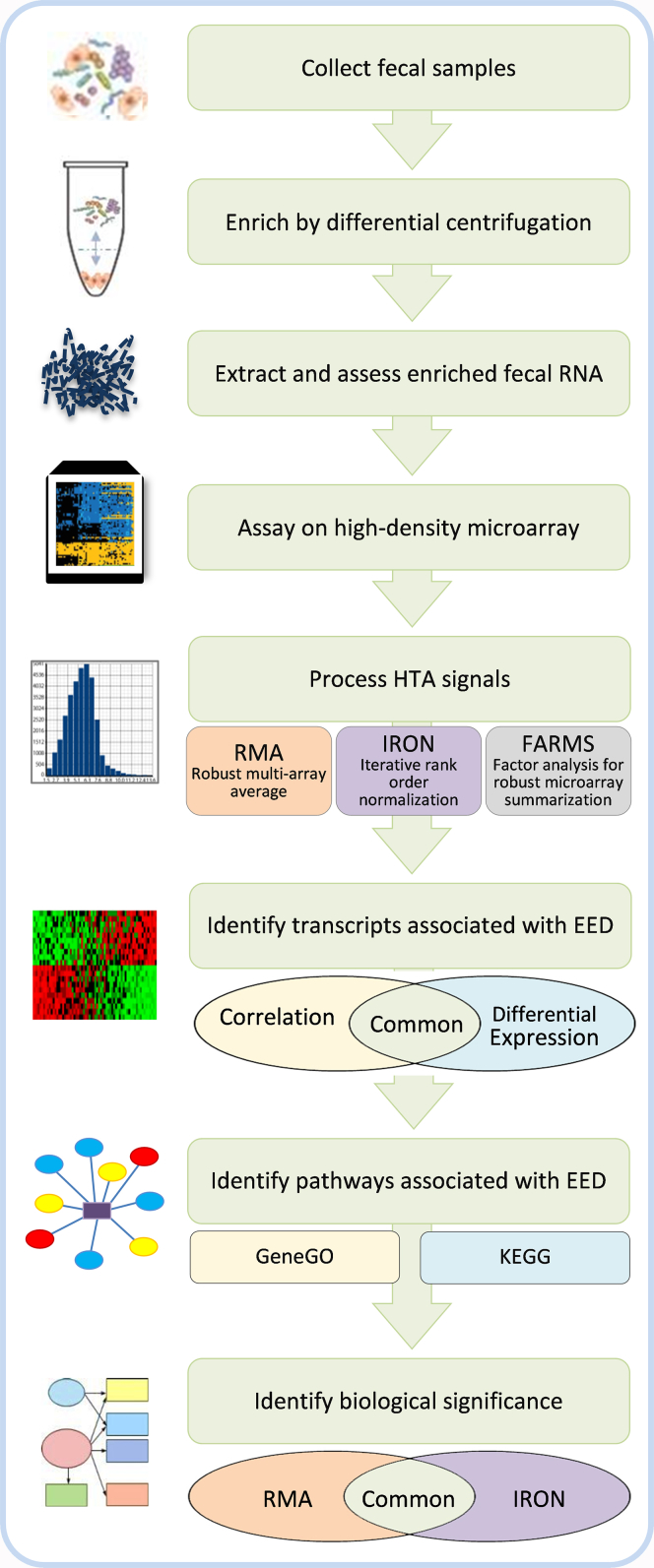
**Schematic flow chart of human fecal transcriptome analysis.** Fecal samples were collected fresh from subjects, immediately flash-frozen in liquid nitrogen, and transported to the laboratory. In the laboratory the cells were suspended in buffer with inert beads and centrifuged at 500*g*. The resulting pellet was kept, resuspended in lysis buffer, and used for total nucleic acids extraction. DNase was added to the nucleic acids mixture, and RNA was separated from the suspension using a bead-based affinity method. The RNA then was amplified and hybridized to a chip containing 25mers covering the entire human genome. The signals corresponding to luminescence for each 25mer were aggregated into genes, and normalized using 3 standard methods. Those transcripts that showed significant correlation with %L, a marker of EED, and differential expression with subsets of increased and normal %L were identified. All transcripts then were used to determine pathway expression for all canonical and KEGG pathways. Transcripts that were correlated with %L, differentially expressed between children with no EED and severe EED, and present in pathways also associated with EED were considered to be of biological significance for EED. HTA, Human Transcriptome Array 2.0 (Affymetrix).

Fresh stools were collected before the dual sugar absorption testing using a small, clean, nonabsorbent, plastic diaper. The stools were transferred immediately to cryovials and flash-frozen in liquid nitrogen. Samples were transferred to a -80°C freezer and transported to Washington University (St. Louis, MO), where they then were processed and analyzed for the human fecal transcriptome as outlined in Figure 1 and detailed later.

### Samples Chosen for Transcriptome Analyses

We chose 259 children for whole-transcriptome analysis on the basis of a mannitol excretion greater than 3%, a total urine volume greater than 15 mL, and a broad distribution of urinary %L values, including normal children. The mannitol was used as a test validation criterion because very small amounts of mannitol absorption indicate very rapid transit intestinal transit times, which distorts the validity of %L as a measure of gut integrity.

### Enriching Fecal Samples for Exfoliated Enterocytes by Differential Centrifugation

Fecal samples were enriched for human cells by differential centrifugation before RNA extraction. Approximately 300–500 mg of frozen stool was transferred to a 15-mL conical tube with 10–15 zirconium/silica beads (2.3 mm) and 3 mL of Hank’s balanced salt solution (Gibco/Life Technologies, Grand Island, NY) with 0.05% Tween-20 (Sigma, St Louis, MO). The samples were vortexed gently for 5 minutes to suspend aggregates. The buffer volume was increased to 10 mL and incubated at 4°C on a rotator for 10 minutes, followed by centrifugation at 1000 rpm (500*g*) for 10 minutes. The supernatant was removed and the pellet was resuspended in 10 mL of Hank’s balanced salt solution/Tween-20 buffer and incubated and centrifuged as before.

### Extracting and Assessing Enriched Fecal RNA

Total fecal nucleic acids were extracted from human-enriched pellets and bacterial-enriched supernatants separately using Specific A protocol on the NucliSENS EasyMAG system (bioMérieux, Durham, NC).^16^, ^17^, ^18^, ^19^ Co-extracted DNA was removed with Baseline-ZERO DNase (Epicentre, Madison, Wisconsin).^20^ Quantitative polymerase chain reaction (qPCR) was performed using TaqMan assays in a droplet digital PCR system (QX100; Bio-Rad Laboratories, Inc, Hercules, CA).^21^, ^22^, ^23^ Human glyceraldehyde 3-phosphate dehydrogenase and bacterial 16S ribosomal RNA (small subunit 16S ribosomal RNA) copies were enumerated to assess the relative human and bacterial RNA content compared with total nucleic acid mass (copies/ng).

### Assaying the Human Fecal Transcriptome on a High-Density Microarray

Human-enriched RNA extracted from differential centrifugation pellets with a minimum of 15 glyceraldehyde 3-phosphate dehydrogenase copies/ng was used for microarray assays. At least 100 ng of DNase-free fecal RNA was amplified with the Ambion WT-plus kit (Ambion/Life Technologies, Grand Island, NY) and hybridized to the GeneChip Human Transcriptome Array 2.0 from Affymetrix (Santa Clara, CA) following the manufacturer’s protocols.^24^ In total, 263 arrays were analyzed, consisting of samples from 259 different individuals and 4 technical replicates.

### Processing Microarray Signals Into Robust Multi-Array Average, Iterative Rank Order Normalization, and Factor Analyses For Robust Microarray Summarization Data Sets

Raw off-scanner microarray intensity data were normalized by 3 standard methods. The 3 methods differ in their assumptions of data distribution and in the method used for background processing, signal normalization, and summarization.

First, robust multi-array average (RMA), the default method, was performed in Affymetrix Expression Console, and involves 3 steps: background correction, quantile normalization, and median-polish summarization.^25^ RMA output includes signal intensity values as well as probe-set level detection *P* values, which filter out individual transcripts with noisy low intensity level.

Second, iterative rank order normalization (IRON) using libaffy version 2.1.5 (http://gene.moffitt.org/libaffy), which consists of RMA background correction, probe-level IRON, the Tukey bi-weight summarization, and a final probe-set or transcript-level IRON.^26^ IRON normalizes through a gradually adjusted subset of invariant features (probe, probe-set, or transcript/gene) in a pair-wise fashion; each individual chip against the reference median chip, the one with the smallest root-mean-square deviation in the data set. IRON output includes only signal intensity values, and detection calls rely upon the RMA method.

Third, factor analyses for robust microarray summarization (FARMS) was performed using the R package FARMS, and does not correct for background but does normalize to quantiles.^27^ Because of an allocation memory issue inherited in the FARMS software, we ran FARMS 10 times for each of 3 randomly grouped subgroups of 259 microarray samples (ie, 30 runs in total). FARMS output includes informative/noninformative calls for genes and probe-sets, in addition to intensity values. The informative/noninformative calls can be used to filter out poorly performing probe-sets and transcripts in the data set.

RMA, IRON, and FARMS data sets each were filtered to exclude microRNA, open reading frame, nonprotein coding, pseudogene, antisense, small nucleolar RNA, and uncharacterized RNA. Transcript clusters for high variable regions of some genes localized on haplotype chromosomes and unplaced contigs such as HLA antigen also were excluded from the analysis. Final analysis thus was performed on 3 transcript-level data sets that each contained log-transformed signal intensities for 18,646 known genes that have a well-annotated official gene symbol.

### Identifying Transcripts Associated With EED by Correlation and Differential Expression

Transcripts correlated to the continuous variable %L were identified by analysis of covariance to 257 normally distributed log_2_-transformed %L values (2 outlier %L values were removed from the total of 259 subjects) using Partek Genomic Suite software, version 6.6 (Partek, Inc, St Louis, MO). Differentially expressed transcripts were identified by analysis of variance between 60 healthy subjects (%L < 0.2) and 42 with severe EED (%L > 0.7) using the R package limma.^28^

### Identifying KEGG and Canonical Pathways Associated With EED

Transcripts that were correlated significantly with %L (analysis of covariance, *P* < .01) were used to identify canonical pathways associated with EED using the GeneGO web tool MetaCore (Thomson Reuters version 6.21, build 66768, Philadelphia, PA).^29^

Fold-change data from differential expression analyses of all transcripts were used to identify enriched KEGG pathways using an R package generally applicable gene set/pathway enrichment.^30^ All significant pathways were defined minimally at *P* < .01, and a false discovery rate less than 0.25.

### Interpreting Biologically Significant Transcripts and Pathways

Biological significance was defined as statistically significant associations between %L and the normalized luminescence measurements in both IRON and RMA data sets. Common transcripts associated with EED were identified by significance in both correlation and differential expression analyses in both IRON and RMA data sets, and then filtered to include only protein-coding genes detected in more than 10% of the 259 arrays. Common pathways associated with EED were defined by enrichment in both RMA and IRON data sets.

Transcripts that were associated with %L also were tested for association with change in HAZ (dHAZ) over the next 3 months, because one of the primary clinical interests of EED is that it is associated with poor linear growth. Growth data were available for 213 of the 259 subjects, and Spearman correlation analysis was performed on 211 normally distributed dHAZ values (2 outlier dHAZ values were removed) using Partek Genomic Suite software, version 6.6 (Partek, Inc).

### Validation of Fecal Transcriptome Results

**Supplementary Figure 1:**
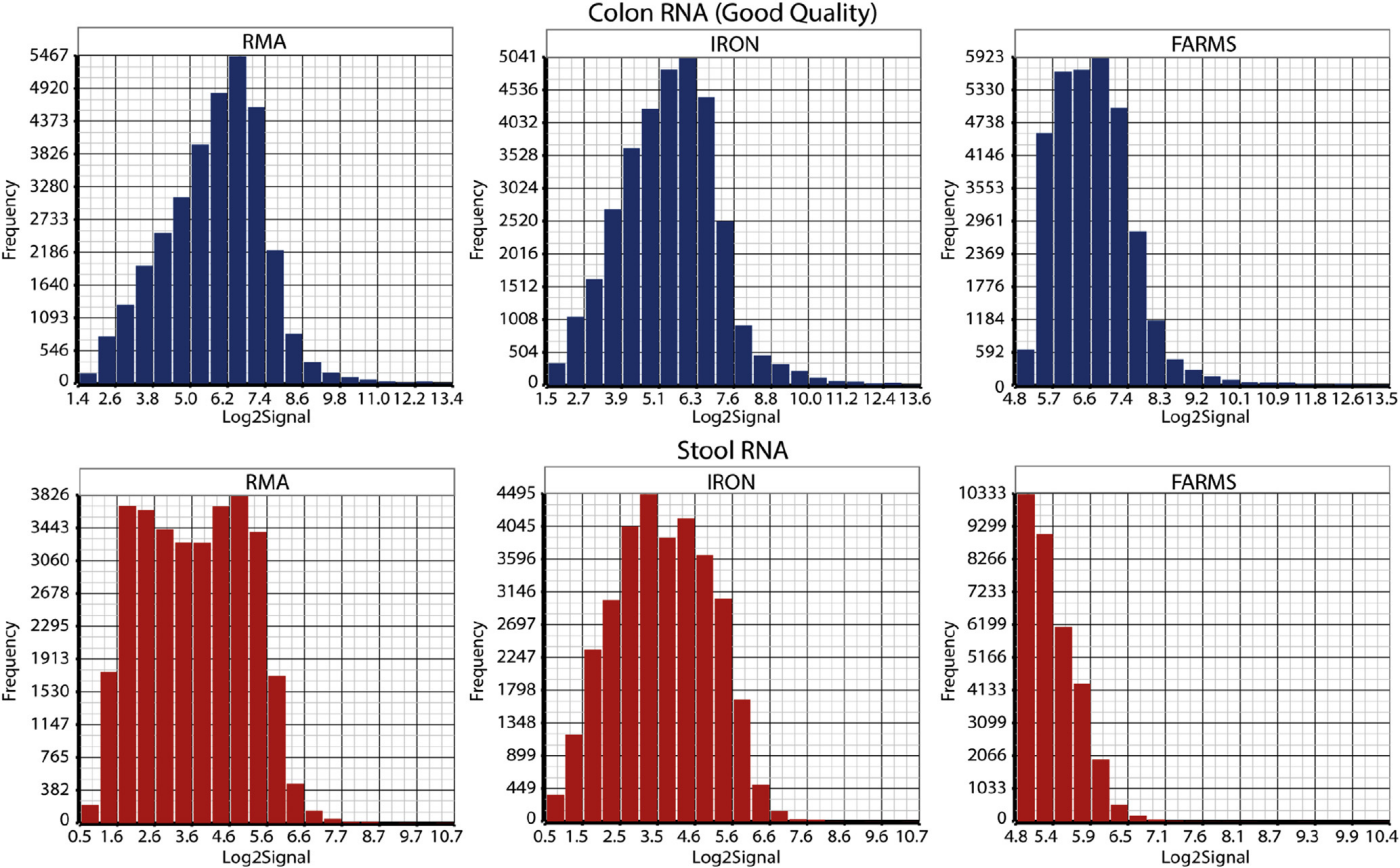
**A comparison of transcript signal intensity and the transcript frequency in samples from colon biopsies and fecal samples.** Note that the distributions of transcripts at a given intensity are similar between both types of samples using 3 different normalization methods, suggesting that these fecal samples are adequate for analyses.

The reproducibility of microarray signals from fecal extractions was validated with the 4 replicate arrays using the Pearson correlation test and illustrated in scatter plots. Furthermore, signal distribution was compared between fecal microarray data and publicly available colon tissue microarray data (Affymetrix Sample Data)^31^ using the Kolmogorov–Smirnov test and shown in histogram (Supplementary Figure 1). Prior qPCR data for 42 genes were available for at least 50 of the 259 subjects, and Pearson correlation analysis was performed between qPCR and transcript level microarray signals for RMA, IRON, and FARMS data sets to validate normalization methods.^21^ Additional qPCR assays were performed on 24 of the 51 transcripts identified by the microarray as associated with EED to validate analysis results.

## Results

### Subjects

**Figure 2:**
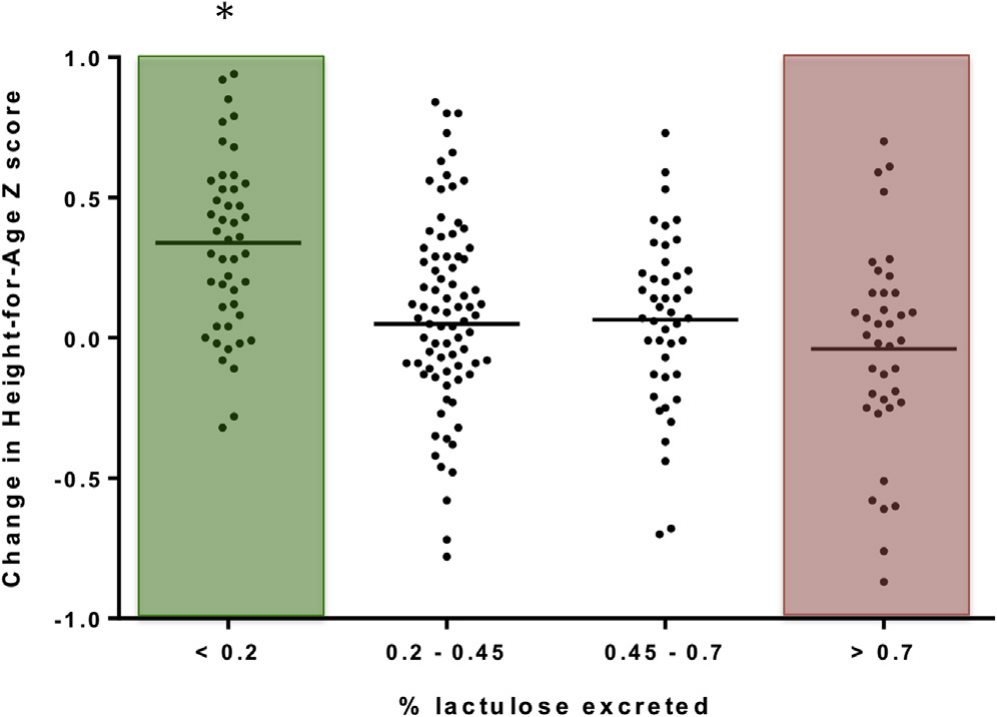
**Association between EED assessed with a dual sugar absorption test and stunting in this population.** Relationship between %L excretion and linear growth, expressed as the change in height-for-age Z-score in the subsequent 3-month period. Data are expressed as means. *Significantly different means from normal children using the Student *t* test with Tukey correction (*P* < .01). The *green bar* represents children without EED showing excellent growth, and the *red bar* represents children with severe EED showing no growth.

A total of 259 rural, asymptomatic, Malawian children at risk for EED were studied (Table 1). The %L was associated with reduced linear growth, expressed as dHAZ in the subsequent 3-month period (Figure 2).

### Human Fecal Messenger RNA Is Reproducibly and Reliably Measured by Microarray

**Figure 3:**
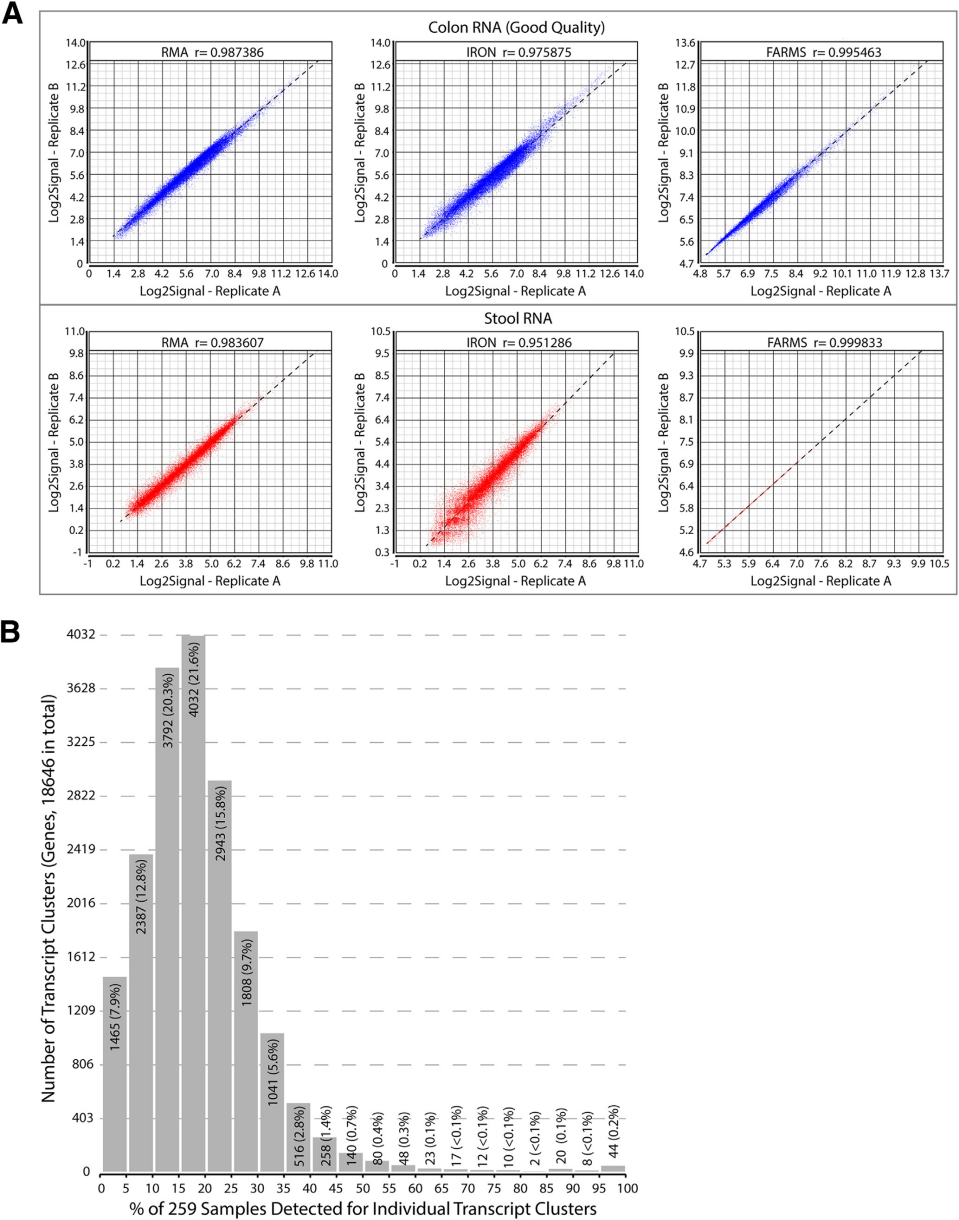
**Reproducibility and detectability of the microarray data using human fecal samples.** (*A*) Scatter plots of technical replicates showing high reproducibility of the microarray data generated using fecal RNAs. These data were quite comparable with that generated using high-quality colon RNA (colon tissue RNAs were adopted from Affymetrix publicly available Sample Data). Note that the FARMS summarized data appears to show a substantial level of compression. Conversely, a somewhat higher degree of variation was noted within the IRON normalized data. (*B*) Histogram of signal detection level showing that microarray technology is reliable in the detection of low copy numbers of fecal RNAs. We calculated the detection of transcript clusters (genes) based on the *P* values reported at probe-set level for intensity data (there were no *P* values reported for the transcript cluster level of intensity data). At first, the total number of detected multiple probe-sets for a given transcript cluster was counted across the entire 259 chips at *P* < .05, then this number was divided by the total number of multiple probe-sets on a chip for this given transcript cluster. Approximately 80% of the 18,646 known genes were detectable in at least 10% of 259 samples.

**Figure 4:**
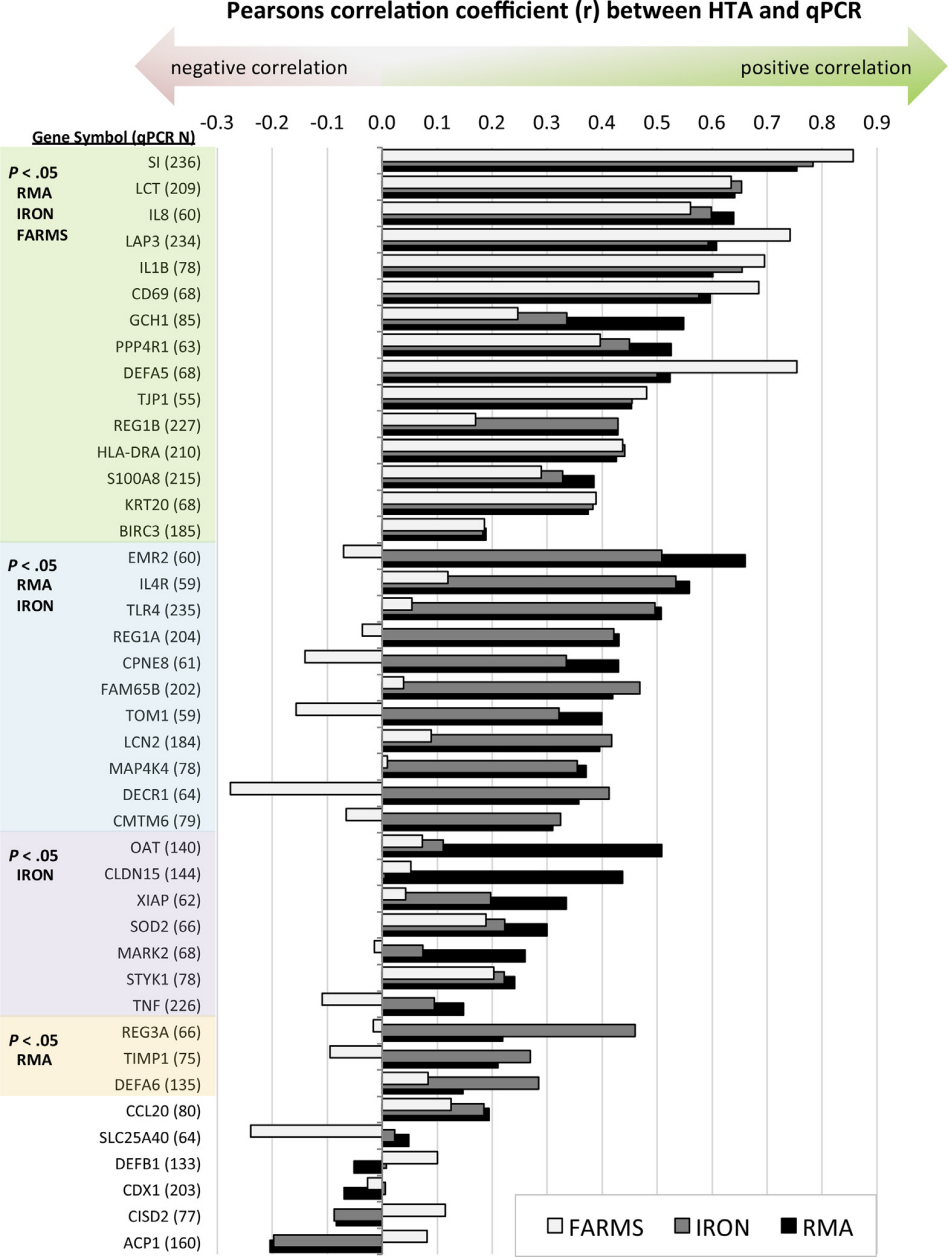
**Correlation between qPCR data and the normalized microarray signals RMA and IRON.** Of the 42 transcripts that were assessed by both microarray and PCR, significant correlations were found in 36 of them by one or more normalization methods. The transcripts were not chosen simply for their association with EED, because some were not associated, but to assess the accuracy of the microarray across the spectrum of protein coding genes.

Expression of all transcripts was highly correlated (Pearson r > 0.95) in replicate arrays regardless of the normalization method (mean ± SD for RMA, 0.98 ± 0.00; IRON, 0.96 ± 0.01; and FARMS, 1.00 ± 0.00), which is comparable with Affymetrix reference microarray data from colon biopsy specimens (Figure 3). Approximately 80% of the 18,646 transcripts were detectable in at least 10% of 259 samples (Figure 3). More similarity between fecal and colon tissue microarrays was observed in the distribution of signal in RMA and IRON data, than in FARMS (Kolmogorov–Smirnov D values: RMA, 0.482; IRON, 0.462; and FARMS, 0.654) (Figure 3). Microarray and qPCR signals also were correlated highly in RMA and IRON normalized data, with significant correlations (*P* < .05) in 79% and 69% of 42 genes tested, respectively, whereas FARMS signals were less correlated to qPCR (Figure 4).

### Microarray Identified Biologically Relevant Transcripts and Pathways Associated With EED

The numbers of transcripts that were correlated significantly to %L (*P* < .01) using either RMA or IRON signal normalized data or those transcripts that were expressed differentially (*P* < .05 and absolute value of fold-change > 1.1) between healthy subjects and those with severe EED are summarized in Table 2. Further interpretation of biological significance focused on those transcripts correlated and expressed differentially in both IRON and RMA normalized data sets because both were well validated by qPCR, and indicated a higher similarity in data distribution between good-quality colon RNA and degraded fecal RNA. Fifty-one common significant transcripts were identified as correlated and expressed differentially in EED (Table 3). The gene symbols are defined and further descriptors of these transcripts are listed in Supplementary Table 1. Twenty-four of these also were tested by qPCR, and the 18 that were detectable all correlated highly to microarray signals (Table 4).

Almost all of the 51 transcripts code for immunologically active proteins, such as IgG or IgE, or for cytokines that modulate the immune response. The molecules encoded include proteins that are made in response to members of various microbial kingdoms, including parasites, bacteria, and viruses (Table 5). Among the 51 transcripts are 6 that code for proteins that affect cell adhesion between epithelial cells. There was a paucity of transcripts that code for structural proteins or enzymes believed to be unique to the small intestine.

**Figure 5:**
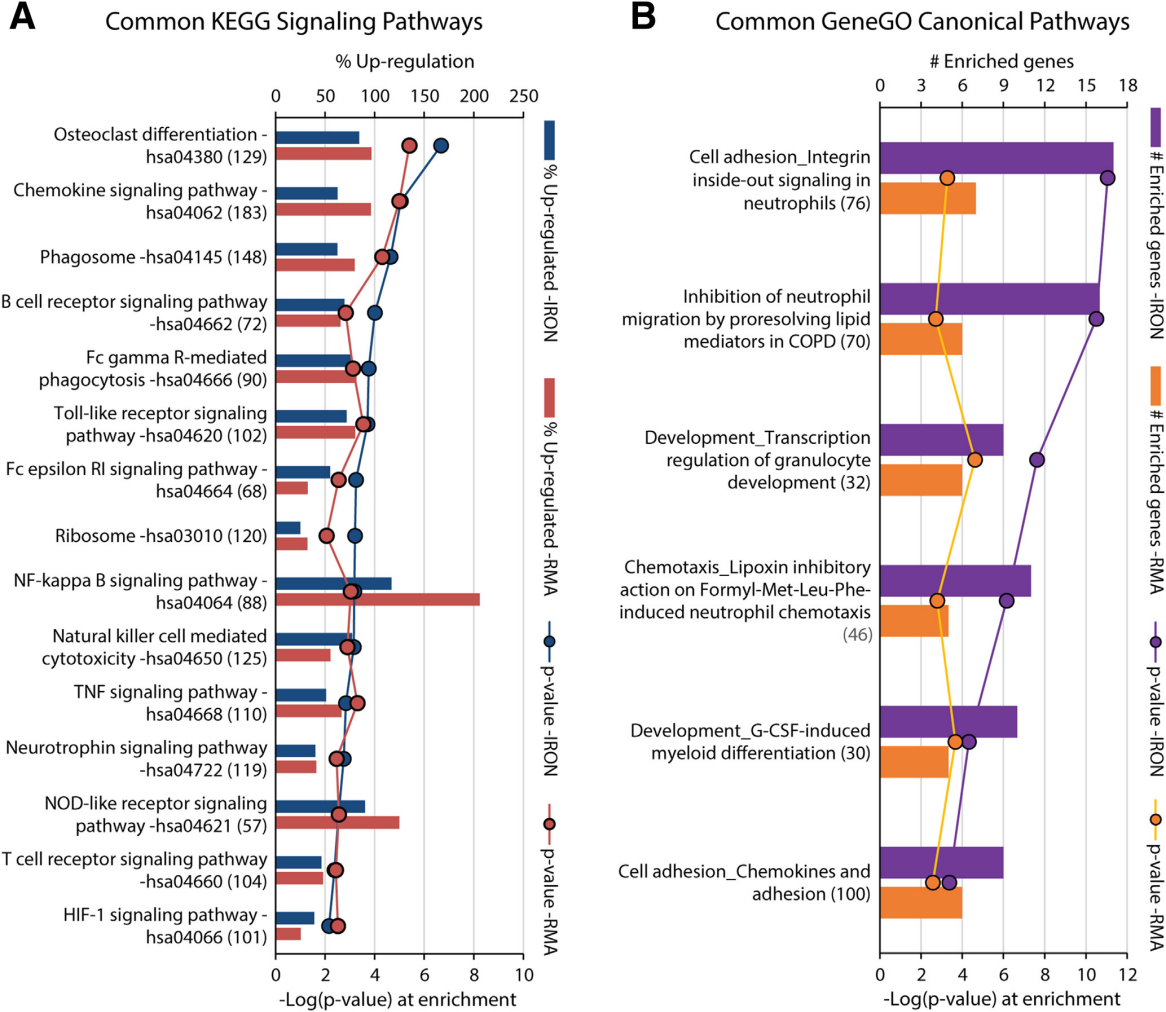
**Association between environmental enteric dysfunction and inflammatory transcripts and pathways.** (*A*) Thirteen common KEGG pathways identified within both RMA- and IRON-normalized microarray intensity data. Numbers after the titles of pathways in parentheses are the number of genes in the data set that were mapped to the given pathways. The significant genes shown are those with an absolute fold-change greater than 1.1 at *P* < .05 in differential analysis. The percentage of up-regulation was calculated using mean fold-change values of significant genes divided by mean fold-change values of nonsignificant genes on the pathways. The -log10 (*P* value) was from pathway analysis, indicating the statistical significance. (*B*) There are 6 common canonical pathways identified using both IRON- and RMA-normalized microarray data. The analysis was performed on genes with a significant correlation between signal intensity and %L value at *P* < .01. The numbers following the titles of pathways are the number of genes in the maps of given pathways. These pathways were significant at *P* < .01 and a false-discovery rate less than 0.25, and the log (*P* values) are shown in the *dotted red lines*. The genes with positive correlation coefficients are shown in gold, and the genes with negative correlation coefficients are shown in blue. COPD, chronic obstructive pulmonary disease; Fc, fragment crystallizable region; HIF-1, hypoxia-inducible factor 1; NF, nuclear factor; NOD, nucleotide-binding oligomerization domain; RI, Fc epsilon RI or high-affinity IgE receptor; TNF, tumor necrosis factor.

**Figure 6:**
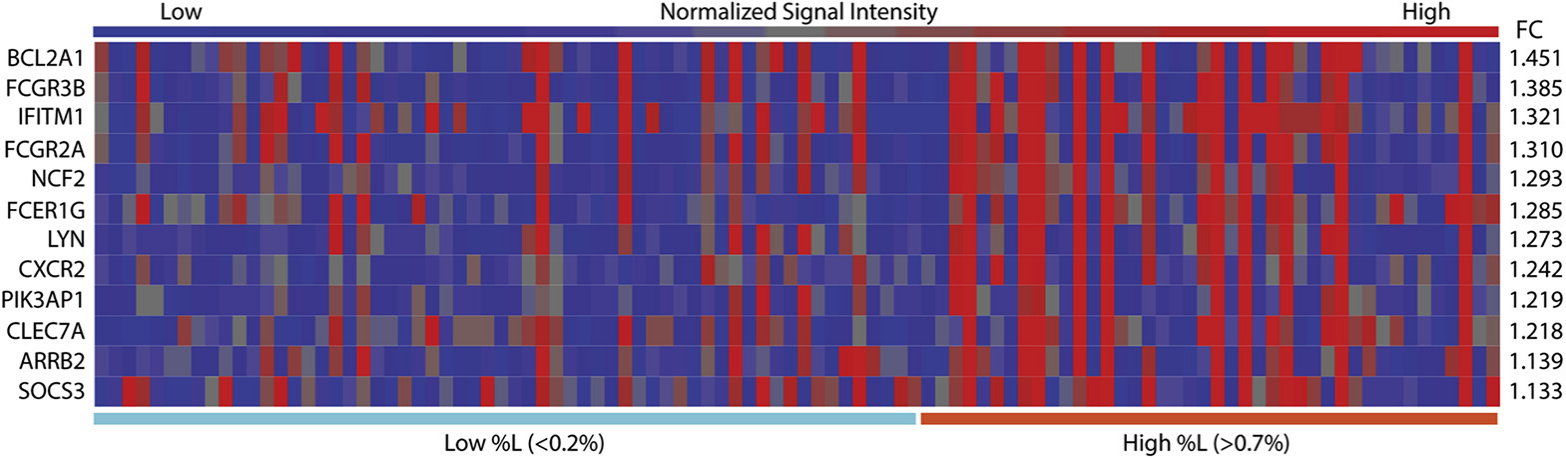
**Heat map for 12 common differentially expressed significant genes, also mapped to significant KEGG pathways, correlated to %L in both IRON-and RMA-normalized microarray expression data.**

Common pathways associated with EED were identified by enrichment in both IRON and RMA data sets, and consist of 6 GeneGO canonical pathways and 15 KEGG signaling pathways (*P* < .01, false-discovery rate < 0.25) that are related predominantly to cell adhesion and immunologic responses (Figure 5 and Supplementary Tables 2 and 3). A subset of 12 of the 51 common transcripts associated with EED map to significantly enriched common KEGG pathways and include chemokines that stimulate T-cell proliferation, Fc fragments of multiple immunoglobulin families, interferon-induced proteins, activators of neutrophils and B cells, and mediators that dampen cellular responses to hormones (Table 6 and Figure 6).

Four mucins (MUC2, MUC4, MUC12, and MUC20), epidermal growth factor receptor, and 3 mitogen-activated protein kinases (MAPK7, MAPK8IP1, and MAPK8IP2), were correlated negatively with %L (*P* < .05 for the Pearson correlation coefficient and the Spearman correlation coefficient using either the IRON or the RMA data set). These 8 proteins, each of which are relevant to mucous biology, are remarkable in that almost all of the other correlations with %L are positive, thereby denoting transcripts that are more abundant with EED. In addition, a negative regulatory transcription factor in goblet cells (recombination signal binding protein for immunoglobulin kappa J region) shows a highly significant correlation with %L (*P* < .01).

Linear growth data over the subsequent 3 months after stool sampling were available from 213 of the 259 children, and expressed as dHAZ to normalize for age. Among the 51 common transcripts associated with %L, 17 also were correlated with dHAZ after normalization with either RMA or IRON (AQP9, CLEC7A, FCGR2A, FCGR3B, IFTM1, IFITM2, IFTM3, LYN, LYZ, MNDA, MSN, NCF2, PLEK, PROK2, S100A8, SAMSN1, and SELL) (Table 3). Among the 12 genes that reside in KEGG pathways that are overexpressed, 6 of these correlated with dHAZ.

## Discussion

In this study the human transcriptome was assessed in individual fecal samples. Previously, host fecal RNA analyses had been performed in samples that were aggregated from similar subjects, to allow for larger amounts of RNA available for analyses^32^, ^33^ or in analyses that targeted specific loci.^21^, ^34^ Our findings suggest that the 25mer, high-density microarray technology coupled with careful fecal specimen collection and a conservative RNA isolation method allows for interrogation of the gut transcriptome. The extensive, whole human transcriptome, nature of the read-outs, and the ability to quantify signals and discern nonrandom pathways lends credence to using transcript capture, rather than sequencing or more tedious and potentially biased specific transcript quantitative PCR, for host organ analysis in stool.

The primary limitation of our methodology was that we did not directly validate the read-outs with transcriptome analyses from biopsy specimens. This would have been impossible in rural Africa, and incompatible with the amplification we used on the fecal specimens. We did not observe many reductions in genes and pathway expression that were associated with EED; this might suggest that our analyses were operating at the edge of the detection limit. Greater sensitivity might have allowed us to detect changes in hormones and cytokines that adversely affect linear growth, although it is not clear that the gut epithelium is the site from which growth-affecting transcriptional responses are generated. We also recognize that out findings are from rural African children consuming a plant-based diet without public sanitation services, and we do not know if these data and this technology can be applied to other populations.

The greatest challenge in this work was to identify a technique that would quantify components of the human fecal transcriptome accurately, given the paucity of specific human messenger RNA (mRNA) in any one sample. Host mRNA is overwhelmed by a much larger population of bacterial transcripts. Further RNA enrichment after extraction, using polyA selection and ribodepletion, did not increase the sensitivity in our microarray method, and risked the loss of target. We assume in addition to being present in low numbers, human host transcripts were likely to be fragmented, having passed through a potentially harsh milieu in the gut. Perhaps by avoiding capture-based enrichment early in the process, and using arrays as the sole, end-preparation, hybridization step before enumeration, we retained more of the fragments of human mRNAs. This yield therefore might produce sufficient human mRNAs in our samples to anneal to the high-density microarray and to provide reproducible transcript-level signals.

The bias introduced by sequence amplification was a limitation of our data. The low copy number present in fecal samples requires amplification to detect them reproducibly by microarray. It is well known that amplification introduces bias on the basis of the probe length, GC content of the probe, and hybridization preference for certain sequences.^35^ These biases prevent us from comparing our data with that from samples that do not require amplification, such as bowel biopsy specimens. However, amplification was applied equally to our entire data set, thus comparisons between samples from children with and without EED are likely to be informative. RNA sequencing and microarray hybridization are methods that both use amplification, and thus both incur these biases.

We used the Affymetrix default data processing and normalization algorithm (RMA-quantile) for our microarray analysis. In light of the presumed degraded nature of fecal RNAs, we also used 2 alternative, but complementary, signal intensity normalization methods (IRON and FARMS) in our analyses. RMA quantile normalization relies on the assumption of Gaussian distributions of data, IRON performs pair-wise intensity normalization without the assumption of Gaussian distribution, and FARMS does not apply background subtraction but summarizes intensities of probe-level based on a linear model with Gaussian noise and Bayesian maximum a posteriori assumptions. FARMS identified just 12 significant transcripts in the differential analysis and no significant pathways in association with EED, suggesting that when samples with low quantities of poor-quality RNA are analyzed, meaningful data are lost when summarizing signals based solely on a linear model. Methods of signal normalization better suited for interpretation of analyses from specimens with low transcript copy numbers need to be developed.

**Figure 7:**
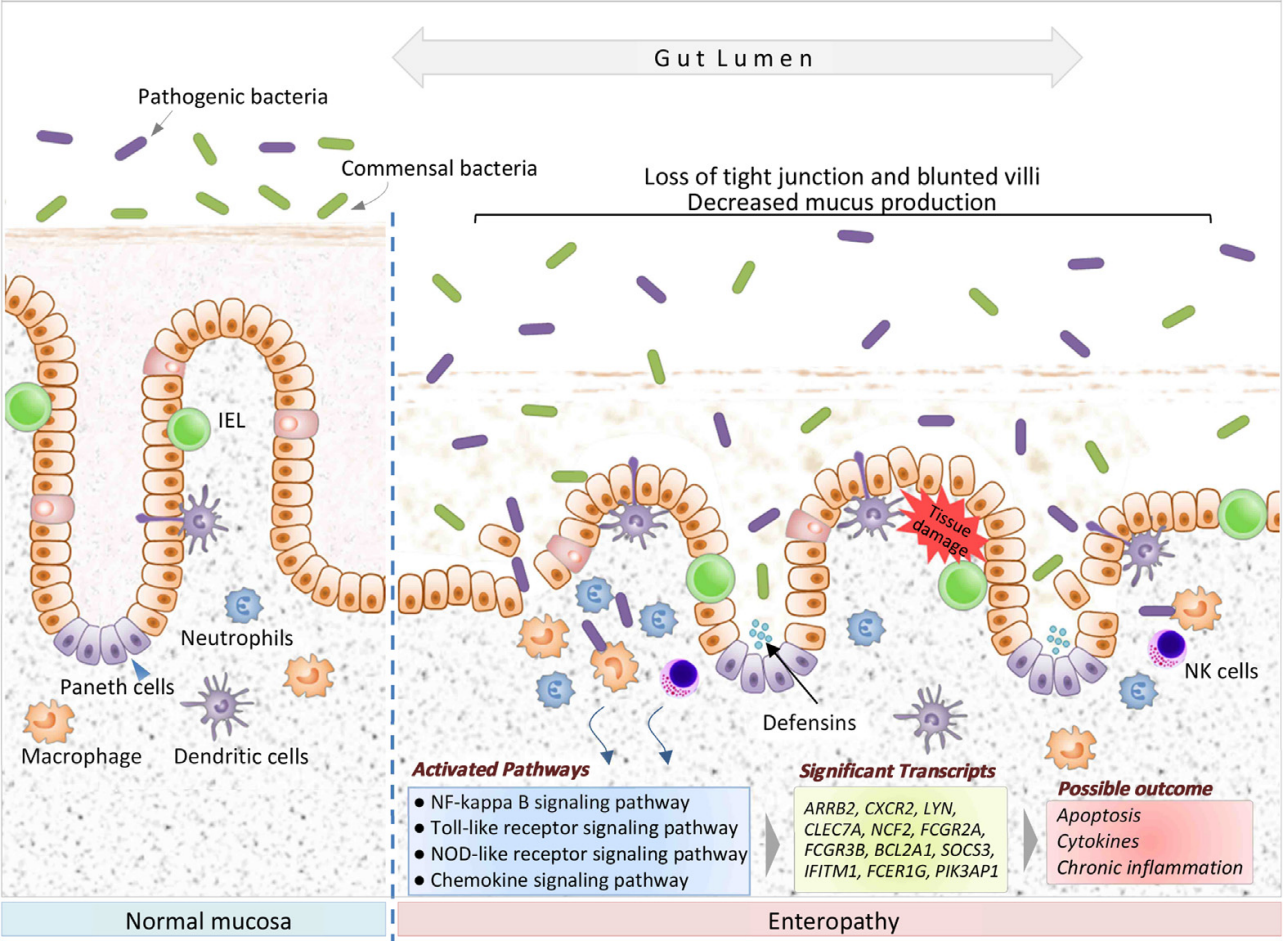
**Summary of the current understanding of the pathobiology of environmental enteric dysfunction.** EED is characterized by increased interaction between epithelial cells and microbes, resulting in changes in the architecture of the small bowel and disruption of the barrier. This is a result of some disruption of the mucous layer, and potentially a dysbiosis between commensal and pathogenic microbes, which include viruses. Multiple immune pathways are chronically activated by this ongoing exposure. Nutrient absorption is reduced owing to the reduction in surface area of the epithelium and damage to the absorptive villi. IEL, intraepithelial lymphocyte; NF, nuclear factor; NK, natural killer; NOD, nucleotide-binding oligomerization domain.

A framework to understand EED emerges from these data, which is summarized in Figure 7. A disrupted mucous layer allows luminal microbes to inflame the mucosa, creating a chronic inflammatory state. The host response is perpetuated by the steady stream of microbes present in the contaminated environments of these rural African children.

A relatively high proportion of the transcripts showing a correlation with EED (Table 6) are associated with myeloid (monocyte, macrophage, dendritic cell, and neutrophil) function. More specifically, a substantial number of these genes are linked to granulocyte colony–stimulating factor (G-CSF) signaling within these and other cell types. CSF 3 receptor is the primary high-affinity receptor for G-CSF and uses the Janus kinase/signal transducer and activator of transcription and V-Yes-1 Yamaguchi Sarcoma Viral Related Oncogene Homolog signal transduction pathways.^36^, ^37^, ^38^, ^39^ Furthermore, SOCS3, BCL2A1, and CXCR2 are induced in response to G-CSF activity. G-CSF pathway activation includes dendritic cell differentiation, neutrophil mobilization, and, more generally, cell survival. G-CSF might protect against infection and also serve survival/repair functions in tissues including the intestine. Given the compromised barrier cell junctions as well as the diffuse villous atrophy observed in EED, it is quite plausible that the G-CSF increase is a compensatory and appropriate response to microbial threat because it augments defense against bacterial translocation and promotes villous repair.

The KEGG pathway analyses suggest that there are immune responses to a diversity of microbes in EED. The role of host–microbial interactions in the duodenum and jejunum currently may be underappreciated because laboratory methods of assessing the bacterial component of the microbiota, predominant in the colon, are so widely used. The expression of genes and the activation of pathways, which promote cell adhesion and phagocytosis, suggest that in EED the host is endeavoring to clean up and repair damaged paracellular junctions in the duodenum and jejunum. The diversity of genes and pathways activated in EED support the speculation that the etiology is multifactorial.^40^

The diversity of immune responses found concurrently in the transcriptome analyses suggests that the mucosa of the small intestine is not shielded from the many microbes in the gut lumen; this is in contrast to gut infections caused by a particular microbe, during which we might expect to see a stronger, more specific immune response. Goblet cells typically respond to inflammatory stimuli with increased mucus secretion. We observed that there was a reduction in transcripts coding for mucin, the protein core of the mucus layer, and the protein kinases that confer the barrier properties to the mucous layer. All of the mucin genes identified with reduced expression are present in the small bowel.^41^, ^42^ The failure of rifaximin to ameliorate EED^15^ suggests that EED is not simply a condition of overgrowth of bacteria in the small intestine, or of infection with organisms susceptible to this antibiotic. Taken together, these data support speculation that EED is the result of, or at least accompanied by, inadequate mucus secretion of the duodenum and jejunum. Our transcriptome findings are summarized in a cartoon in Figure 7. In addition, penetration of the epithelial paracellular junctions by nonviable vesicles secreted by bacteria evokes a strong inflammatory response, and this possibility also should be considered in EED.^43^

Of the 51 transcripts in which there is increased expression in EED, 29 are reported to respond to viral infection. We speculate that virus presence and infection might play a role in EED, and as our ability to characterize and understand the role of viruses in the duodenum and jejunum increases, researchers should investigate this possibility further. Indeed, children from resource-poor regions have a much more diverse intestinal virome than those from high-income settings, and our data suggest that the childhood gut might be responding to these agents.^44^

The association of 33%–50% of the EED-associated transcripts with subsequent linear growth is consistent given the association with %L and linear growth in this population.

Finally, the list of transcripts differentially expressed in EED may provide direction to those seeking a better biomarker for this elusive, formidable, scourge of children in the developing world. The opportunity to interrogate the host transcriptome of the gastrointestinal tract through a fecal specimen may be exploited to develop biomarkers for other inflammatory and carcinogenic diseases of the gut in the future. Our data also showed the feasibility and potential superiority of direct from extract (ie, does not require additional treatment or manipulation as in previous protocols) immobilization of RNA on a support platform, on which transcripts can be quantified directly. It is possible that this methodology optimizes yield, and purity, of the molecules of interest (ie, human mRNAs) in the complex milieu of the fecal biomass.

## Figures and Tables

**Table 1:** Characteristics of Malawian Study Children at Risk for Environmental Enteric Dysfunction

Characteristic	Mean ± SD or N (%)
Male sex	134 (52)
Age, *mo*	30.1 ± 11.2
Weight-for-height, z score	-0.1 ± 0.9
Height-for-age, z score	-2.3 ± 1.2
Caretaker is mother	249 (96)
Father is alive	256 (99)
Siblings	3.2 ± 1.9
Individuals who sleep in the same room as the child	3.1 ± 1.3
Home with a metal roof	67 (26)
Family owns a bicycle	147 (57)
Animals sleep in the house	118 (46)
Water from a clean source	206 (80)
Child uses pit latrine	125 (48)
Child has HIV infection	3 (1)
Mother reports child has loose stools	4 (2)
Lactulose:mannitol ratio	0.3 ± 0.2
Urinary lactulose, % dose administered	0.4 ± 0.3
Urinary mannitol, % dose administered	6.7 ± 3.0
EED severity	
Healthy	60 (23)
Intermediate EED	157 (61)
Severe EED	42 (16)

HIV, human immunodeficiency virus.

**Table 2:** Transcripts and Pathways Associated With Environmental Enteric Dysfunction Resulting From 3 Normalization and Summarization Methods

Normalization method	%L correlated transcripts	Differentially expressed transcripts	Enriched KEGG pathways	Enriched canonical pathways
Criteria for inclusion	ANCOVA *P* < .01	ANOVA *P* < .05 and fold-change > 1.1	GAGE *P* < .01 and FDR < 0.25	MetaCore *P* < .01 and FDR < 0.25
RMA	637 (3.42%)	141 (0.76%)	17 (8.95%)	8 (4.97%)
IRON	667 (3.58%)	388 (2.08%)	19 (10.00%)	46 (28.57%)
FARMS	81 (0.43%)	12 (0.06%)	0 (0%)	17 (10.56%)

ANCOVA, analysis of covariance; ANOVA, analysis of variance; FDR, false-discovery rate; GAGE, generally applicable gene set enrichment for pathway analysis; MetaCore, integrated software for functional analysis.

**Table 3:** Transcripts Associated With EED

Gene symbol	Detected in 259	Differential expression, healthy vs severe EED		Pearson correlation to %L		Spearman correlation to dHAZ	
FC/*P* value (RMA)	FC/*P* value (IRON)	r/*P* value (RMA)	r/*P* value (IRON)	rho/*P* value (RMA)	rho/*P* value (IRON)
ACSL1	26%	1.16/.006	1.27/.003	0.18/.004	0.19/.002	-0.05/.463	-0.05/.480
AMICA1	23%	1.12/.002	1.22/.000	0.20/.001	0.23/.000	-0.10/.148	-0.09/.218
AQP9	22%	1.33/.003	1.39/.004	0.17/.006	0.17/.007	-0.14/.038	-0.12/.083
ARRB2	42%	1.12/.009	1.14/.008	0.18/.003	0.18/.004	-0.04/.590	-0.07/.337
BCL2A1	26%	1.44/.003	1.45/.008	0.18/.004	0.18/.003	-0.09/.216	-0.12/.087
BCL6	21%	1.13/.005	1.16/.010	0.17/.007	0.18/.004	-0.07/.280	-0.10/.148
BIN2	30%	1.15/.002	1.23/.002	0.18/.004	0.20/.001	-0.10/.155	-0.06/.423
CD53	20%	1.20/.002	1.34/.001	0.17/.006	0.20/.001	-0.13/.063	-0.09/.186
CLEC7A	19%	1.11/.001	1.22/.001	0.20/.001	0.21/.001	-0.16/.018	-0.15/.033
CR1	16%	1.13/.001	1.23/.000	0.21/.001	0.24/.000	-0.06/.381	-0.02/.770
CSF2RB	18%	1.10/.010	1.14/.006	0.16/.010	0.18/.004	-0.07/.311	-0.09/.180
CSF3R	23%	1.12/.001	1.19/.000	0.22/.000	0.25/.000	-0.09/.176	-0.13/.059
CST7	36%	1.14/.001	1.18/.003	0.19/.002	0.18/.004	-0.10/.148	-0.12/.089
CXCR2	21%	1.16/.002	1.24/.001	0.17/.005	0.19/.002	-0.04/.529	-0.10/.165
FAM157A	24%	1.17/.018	1.22/.022	0.17/.005	0.16/.008	-0.04/.533	-0.03/.639
FAM157B	26%	1.13/.003	1.25/.001	0.19/.002	0.21/.001	0.03/.683	0.04/.579
FCER1G	24%	1.17/.002	1.29/.001	0.19/.002	0.21/.001	-0.08/.229	-0.12/.082
FCGR1B	19%	1.16/.003	1.25/.003	0.19/.002	0.18/.004	-0.07/.320	-0.05/.458
FCGR2A	25%	1.19/.002	1.31/.001	0.19/.002	0.19/.002	-0.15/.035	-0.14/.049
FCGR3B	23%	1.29/.002	1.39/.001	0.19/.002	0.18/.003	-0.18/.008	-0.20/.003
FFAR2	15%	1.33/.001	1.38/.002	0.19/.002	0.18/.005	-0.07/.311	-0.01/.887
FPR1	11%	1.24/.000	1.31/.000	0.19/.002	0.20/.002	-0.11/.116	-0.08/.240
GPR84	20%	1.11/.006	1.15/.015	0.16/.010	0.16/.009	-0.11/.097	-0.10/.143
IFI30	24%	1.24/.002	1.30/.002	0.20/.001	0.20/.001	-0.06/.378	-0.05/.461
IFITM1	41%	1.21/.001	1.32/.002	0.19/.002	0.20/.001	-0.19/.006	-0.18/.008
IFITM2	48%	1.42/.001	1.51/.001	0.20/.001	0.20/.001	-0.14/.041	-0.15/.024
IFITM3	45%	1.31/.001	1.38/.001	0.19/.003	0.20/.001	-0.11/.097	-0.16/.023
IL1RN	18%	1.14/.001	1.24/.002	0.17/.007	0.17/.005	-0.13/.057	-0.11/.106
LAPTM5	13%	1.24/.002	1.28/.004	0.18/.003	0.17/.005	-0.06/.423	-0.07/.328
LCP1	16%	1.17/.003	1.25/.003	0.19/.002	0.19/.002	-0.10/.153	-0.09/.201
LYN	26%	1.17/.008	1.27/.005	0.19/.003	0.19/.002	-0.14/.046	-0.15/.031
LYZ	22%	1.27/.000	1.41/.001	0.18/.004	0.17/.007	-0.10/.134	-0.16/.023
MNDA	30%	1.33/.004	1.52/.004	0.17/.007	0.17/.007	-0.18/.009	-0.18/.009
MSN	29%	1.11/.004	1.18/.002	0.17/.006	0.20/.001	-0.11/.114	-0.15/.030
NCF2	15%	1.20/.000	1.29/.000	0.24/.000	0.22/.000	-0.12/.080	-0.15/.034
NOP10	46%	1.12/.007	1.21/.001	0.17/.007	0.21/.001	0.00/.981	-0.13/.062
OR52D1	18%	1.13/.001	1.15/.022	0.24/.000	0.18/.005	-0.08/.278	-0.06/.401
PIK3AP1	24%	1.13/.004	1.22/.001	0.19/.002	0.21/.001	-0.01/.850	-0.06/.351
PLEK	39%	1.67/.003	1.56/.010	0.18/.003	0.17/.007	-0.14/.042	-0.11/.117
PROK2	26%	1.27/.000	1.34/.001	0.19/.003	0.18/.004	-0.08/.249	-0.14/.040
S100A12	10%	1.22/.006	1.31/.008	0.18/.004	0.17/.005	-0.10/.143	-0.08/.276
S100A8	27%	1.17/.004	1.39/.002	0.17/.008	0.18/.004	-0.13/.051	-0.14/.045
SAMSN1	24%	1.14/.022	1.30/.001	0.17/.005	0.21/.001	-0.12/.095	-0.18/.008
SDCBP	25%	1.20/.006	1.36/.009	0.19/.003	0.17/.006	-0.10/.148	-0.11/.118
SELL	19%	1.24/.001	1.43/.000	0.19/.002	0.24/.000	-0.15/.032	-0.16/.017
SLC2A3	26%	1.16/.006	1.25/.005	0.18/.004	0.18/.004	-0.12/.085	-0.13/.070
SOCS3	13%	1.12/.003	1.13/.005	0.18/.003	0.18/.004	-0.03/.627	-0.08/.277
SORL1	24%	1.17/.002	1.23/.005	0.20/.001	0.19/.002	-0.11/.101	-0.12/.094
TAGAP	23%	1.15/.007	1.24/.007	0.17/.008	0.19/.002	-0.10/.144	-0.07/.319
VNN2	18%	1.15/.002	1.25/.002	0.20/.001	0.21/.001	-0.11/.101	-0.12/.092
XPO6	21%	1.16/.002	1.21/.007	0.20/.002	0.19/.002	-0.04/.591	-0.06/.372

NOTE. Transcripts with differential expression: healthy %L less than 0.2 vs severe EED %L greater than 0.7; Pearson correlations with %L; and Spearman correlation with change in height-for-age Z score in the subsequent 3-month period.

**Table 4:** Validation of 18 Common Transcripts Associated With EED by Quantitative PCR: ddPCR

Target	N (ddPCR)	Correlation between droplet digital PCR and microarray (IRON)		Correlation between droplet digital PCR and microarray (RMA)	
Spearman correlation coefficient/significance (2-tailed)	Pearson correlation coefficient/significance (2-tailed)	Spearman correlation coefficient/significance (2-tailed)	Pearson correlation coefficient/significance (2-tailed)
ACSL1	39	0.833/0.000	0.559/0.000	0.818/0.000	0.607/0.000
AQP9	39	0.699/0.000	0.636/0.000	0.725/0.000	0.669/0.000
BCL2A1	39	0.726/0.000	0.726/0.000	0.743/0.000	0.763/0.000
CD53	39	0.706/0.000	0.619/0.000	0.753/0.000	0.648/0.000
CSF3R	24	0.785/0.000	0.654/0.001	0.746/0.000	0.798/0.000
IFI30	39	0.698/0.000	0.467/0.003	0.701/0.000	0.478/0.002
IL1RN	36	0.801/0.000	0.743/0.000	0.739/0.000	0.632/0.000
LAPTM5	39	0.718/0.000	0.565/0.000	0.690/0.000	0.644/0.000
LCP1	33	0.835/0.000	0.583/0.000	0.837/0.000	0.584/0.000
LYN	39	0.833/0.000	0.531/0.001	0.843/0.000	0.532/0.000
LYZ	39	0.684/0.000	0.501/0.001	0.672/0.000	0.535/0.000
MNDA	25	0.775/0.000	0.513/0.009	0.778/0.000	0.496/0.012
PIK3AP1	39	0.802/0.000	0.741/0.000	0.843/0.000	0.714/0.000
PLEK	39	0.883/0.000	0.615/0.000	0.885/0.000	0.666/0.000
SELL	41	0.808/0.000	0.580/0.000	0.802/0.000	0.611/0.000
SLC2A3	33	0.761/0.000	0.557/0.001	0.710/0.000	0.626/0.000
SORL1	33	0.648/0.000	0.569/0.001	0.694/0.000	0.656/0.000
TAGAP	39	0.811/0.000	0.517/0.001	0.841/0.000	0.57/0.000

NOTE. Twenty-four targets were chosen for qPCR validation from the 51 transcripts listed in Table 3. Of the 24 targets, 18 were detectable. Pearson and Spearman correlations for all 18 were highly significant.

**Table 5:** Selected Functions of the 51 Transcripts Associated With EED

Gene symbol	Description	Cell adhesion	Viral response	Bacterial response	Parasite response	Fungal response	Localized to small intestine
ACSL1	Acyl-CoA synthetase long-chain family member 1						
AMICA1	Adhesion molecule, interacts with CXADR antigen 1	X	X	X			
AQP9	Aquaporin 9			X			
ARRB2	Arrestin, β 2		X				
BCL2A1	BCL2-related protein A1		X	X			
BCL6	B-cell CLL/lymphoma 6		X				
BIN2	Bridging integrator 2			X			
CD53	CD53 molecule		X	X	X	X	
CLEC7A	C-type lectin domain family 7, member A					X	
CR1	Complement component (3b/4b) receptor 1 (Knops blood group)		X	X	X		
CSF2RB	Colony-stimulating factor 2 receptor, β, low-affinity		X	X			
CSF3R	Colony-stimulating factor 3 receptor (granulocyte)	X	X	X			
CST7	Cystatin F (leukocystatin)		X	X			
CXCR2	Chemokine (C-X-C motif) receptor 2		X	X	X		
FAM157A	Family with sequence similarity 157, member A						
FAM157B	Family with sequence similarity 157, member B						
FCER1G	Fc fragment of IgE, high-affinity I, receptor for; γ polypeptide				X		
FCGR1B	Fc fragment of IgG, high-affinity Ib, receptor (CD64)		X	X			
FCGR2A	Fc fragment of IgG, low-affinity IIa, receptor (CD32)		X	X			
FCGR3B	Fc fragment of IgG, low-affinity IIIb, receptor (CD16b)		X	X			
FFAR2	Free fatty acid receptor 2		X	X			X
FPR1	Formyl peptide receptor 1			X			
GPR84	G-protein–coupled receptor 84		X	X	X		
IFI30	Interferon, γ-inducible protein 30		X				
IFITM1	Interferon-induced transmembrane protein 1		X				
IFITM2	Interferon-induced transmembrane protein 2		X				
IFITM3	Interferon-induced transmembrane protein 3		X				
IL1RN	Interleukin 1–receptor antagonist		X	X			
LAPTM5	Lysosomal protein transmembrane 5			X			
LCP1	Lymphocyte cytosolic protein 1 (L-plastin)		X	X			X
LYN	V-yes-1 Yamaguchi sarcoma viral-related oncogene homolog		X	X	X		
LYZ	Lysozyme			X			
MNDA	Myeloid cell nuclear differentiation antigen		X				
MSN	Moesin	X	X				
NCF2	Neutrophil cytosolic factor 2			X			
NOP10	NOP10 ribonucleoprotein						
OR52D1	Olfactory receptor, family 52, subfamily D, member 1						
PIK3AP1	Phosphoinositide-3-kinase adaptor protein 1		X	X			
PLEK	Pleckstrin						
PROK2	Prokineticin 2						X
S100A12	S100 calcium binding protein A12		X	X	X	X	
S100A8	S100 calcium binding protein A8		X	X	X	X	
SAMSN1	SAM domain, SH3 domain and nuclear localization signals 1		X	X			
SDCBP	Syndecan binding protein (syntenin)	X					
SELL	Selectin L	X			X		
SLC2A3	Solute carrier family 2 (facilitated glucose transporter), member 3						
SOCS3	Suppressor of cytokine signaling 3		X	X	X	X	
SORL1	Sortilin-related receptor, L (DLR class) A repeats containing						
TAGAP	T-cell activation RhoGTPase activating protein						X
VNN2	Vanin 2	X					
XPO6	Exportin 6						

**Table 6:** Transcripts Correlated With Environmental Enteric Dysfunction by Two Normalization Methods That Also Map to KEGG Pathways

Gene symbol	Gene description	Pathway category	r/*P* value (IRON)	r/*P* value (RMA)
BCL2A1	BCL2-related protein A1: retards apoptosis induced by interleukin 3 deprivation	Physiologic stress	0.184/.003	0.181/.003
FCGR3B	Fc fragment of IgG, low-affinity IIIb, receptor (CD16b): binds to Fc region of immunoglobulins gamma. Low-affinity receptor. Binds complexed or aggregated IgG and also monomeric IgG. Not capable of mediating antibody-dependent cytotoxicity and phagocytosis	Phagocytosis	0.184/.003	0.189/.002
IFITM1	Interferon-induced transmembrane protein 1: antiviral protein that inhibits the entry of viruses to the host cell cytoplasm, permitting endocytosis, but preventing subsequent viral fusion and release of viral contents into the cytosol. Active against multiple viruses	Response to viral invasion	0.198/.001	0.188/.002
FCGR2A	Fc fragment of IgG, low-affinity IIa, receptor (CD32): binds to the Fc region of IgG. Binds to IgG and initiates cellular responses against pathogens and soluble antigens	Phagocytosis	0.190/.002	0.189/.002
NCF2	Neutrophil cytosolic factor 2: required for activation of the latent NADPH oxidase	Phagocytosis	0.217/.001	0.237/.001
FCER1G	Fc fragment of IgE, high-affinity I, receptor for; γ polypeptide: the high-affinity IgE receptor is a key molecule involved in allergic reactions	Response to viral invasion	0.214/.001	0.190/.002
LYN	V-yes-1 Yamaguchi sarcoma viral-related oncogene homolog: nonreceptor tyrosine-protein kinase. Plays an important role in the regulation of B-cell differentiation, proliferation, survival, and apoptosis, and is important for immune self-tolerance	Response to infection	0.192/.002	0.187/.003
CXCR2	Chemokine (C-X-C motif) receptor 2: integral membrane proteins that specifically bind and respond to cytokines of the CXC chemokine family. Receptor for interleukin 8, which is a powerful neutrophil chemotactic factor. Binds to interleukin 8 with high affinity	Physiologic stress	0.190/.002	0.173/.005
PIK3AP1	Phosphoinositide-3-kinase adaptor protein 1: signaling adapter that contributes to B-cell development, controls excessive inflammatory cytokine production by linking TLR signaling to PI3K activation	Response to viral invasion	0.211/.001	0.188/.002
CLEC7A	C-type lectin domain family 7, member A: functions as a pattern-recognition receptor for a variety of β-1,3-linked and β-1,6-linked glucans, such as cell wall constituents from pathogenic bacteria and fungi, and plays a role in innate immune response. Stimulates T-cell proliferation	Phagocytosis	0.212/.001	0.201/.001
ARRB2	Arrestin, β 2: functions in regulating agonist-mediated desensitization of G-protein–coupled receptor and cause specific dampening of cellular responses to stimuli such as hormones, neurotransmitters, or sensory signals	Physiologic stress	0.180/.004	0.182/.003
SOCS3	Suppressor of cytokine signaling 3: negative regulator of JAK/STAT pathway. Inhibits cytokine signal transduction by binding to tyrosine kinase receptors including gp130, LIF, erythropoietin, insulin, interleukin 12, G-CSF, and leptin receptors	Physiologic stress	0.181/.003	0.182/.003

JAK/STAT, Janus kinase/signal transducer and activator of transcription; LIF, leukemia inhibitory factor; NADPH, reduced nicotinamide adenine dinucleotide phosphate; PI3K, phosphoinositide 3-kinase; TLR, Toll-like receptor.
